# Assessing Sleep Quality and Influencing Factors Among Medical Undergraduates: A Cross-Sectional Study

**DOI:** 10.7759/cureus.78919

**Published:** 2025-02-12

**Authors:** Premsagar J Vasava, Pratik K Jasani, Kishor M Sochaliya, Milindkumar H Makwana, Mayank R Kapadiya, Saif Ali S Kadri, Vijay G Ahir, Jay H Nimavat

**Affiliations:** 1 Department of Community Medicine, C.U. Shah Medical College and Hospital, Surendranagar, IND

**Keywords:** body mass index(bmi), pittsburg sleep quality index (psqi), quality of sleep, stress, under graduate medical students

## Abstract

Background: Sleep is a biological process that is essential for life. Sleep quality refers to an individual's overall satisfaction with their sleep experience, encompassing factors such as ease of falling asleep, ability to stay asleep, total sleep duration, and the sense of refreshment upon waking*.* Undergraduate medical students frequently struggle with good-quality sleep. Poor sleep quality negatively impacts academic performance, behavioural issues, emotional issues, bad emotional status, and also increases the risk of alcohol, smoking habits and psychiatric disorders.

Objective: This study aims to assess the quality of sleep and to find out its influencing factors among undergraduate medical students.

Methods: An observational survey was carried out at C.U. Shah Medical College, Surendranagar, India between 1^st^ June to 31^st^ August 2024 among undergraduate medical students. Study participants were selected using convenient sampling technique with their consent and the final sample size was 300. Data was collected by a pre-tested, semi-structured questionnaire comprised of the Pittsburgh Sleep Quality Index (PSQI) scale, sociodemographic profile and habitual practices of students through a Google form. Data was analysed with the help of SPSS version 26 (IBM Corp., Armonk, NY, USA).

Results: The study showed that about 34% of study participants had poor quality of sleep. A statistically significant association was found between quality of sleep and sociodemographic variables like phase of studying for Bachelor of Medicine, Bachelor of Surgery (MBBS) (p=0.004) and Body Mass Index (p=0.04). There was a strong statistical association found between perceived stress during the last month and quality of sleep (p<0.000).

Conclusions: Sleep quality was poor in 34.0% of study participants. BMI, phase of studying MBBS, and perceived stress during the last month were major determinants affecting the quality of sleep among medical students. Undergraduate students should adopt stress management techniques and maintain a healthy BMI to improve sleep quality during their MBBS studies.

## Introduction

Sleep is a biological process that is essential for life. Sleep quality is defined as "one's satisfaction of the sleep experience, integrating aspects of sleep initiation, sleep maintenance, sleep quantity, and refreshment upon awakening" [1]. Sleep quality has a strong connection to several well-being indicators as well as physical and mental health [2]. Adequate sleep is essential for physical and mental well-being as well as appropriate neurocognitive and psychomotor functioning of an individual [3].

Over the past few years, issues with sleep and its related problems have gotten more attention. The core reason for this interest is the awareness that fatigue and sleeplessness have become prevalent across the population [4]. Young doctors in their early years often lack sleep since they stay up late in medical college to study for exams, which is followed by extended stays in hospitals. Students' health and way of life are being impacted by the rising stress levels they are experiencing as well as the demanding schedules at the hospital [5].

Undergraduate medical students frequently struggle with good-quality sleep [6]. The present scenario is that college students don't get enough sleep [7]. Poor sleep quality negatively impacts academic performance, behavioural issues, emotional issues, bad emotional status, and increases the risk of alcohol, smoking habits and psychiatric disorders [8]. 

During the academic years of college, students have come across many difficulties, including adjusting to a different teaching-learning structure, losing their social network, and changing their lifestyles [9]. Their sleep quality suffered as a result, and their stress, anxiety, and depressive symptoms were worsened [10]. In a Chinese study, 19% of medical students were found to have poor sleep quality as assessed by the Pittsburgh Sleep Quality Index (PSQI), with differences seen between years of study but not between genders. Another study of Chinese medical students reported that more than 90% of the undergraduates had experienced excessive sleepiness in class, with more males than females affected. About 70% of Hong Kong medical students self-reported sleep deprivation, confirmed by objective Actiwatch (Philips, Amsterdam, The Netherlands) recordings, with no significant gender and age differences [11].

Very few studies have been done so far to provide insight into the sleep health of medical undergraduates. So, this study aimed to assess the quality of sleep and determine its influencing factors among undergraduates studying at a medical college in Surendranagar district, Gujarat, India.

## Materials and methods

A cross-sectional survey was executed among medical undergraduates of C. U. Shah Medical College, a tertiary care teaching hospital of Surendranagar district, Gujarat between June to August 2024. This institute enrolls 100 undergraduate students annually in its Bachelor of Medicine, Bachelor of Surgery (MBBS) course. All undergraduate medical students from Phase I to Phase III part II were encouraged to participate in this research. The total target population for this study consisted of 400 students, as the college enrolls 100 students each year, with a cumulative total of 400 students after Phase III part II. Out of these, 300 provided informed consent and met the inclusion criteria for participation in the study. So, the final sample size for the study was 300. Before initiating the study, approval from the Institutional Ethical Committee was acquired with approval No. CUSMC/IEC(HR)/RP/29/2024/Final approval/267/2024. A self-administered survey form was used to obtain data. Pilot research was carried out among 30 undergraduate students and the survey form had been modified accordingly. The survey form included sociodemographic information, the PSQI scale and habitual practice of medical students. Socio-demographic characteristics included age, gender, year of the study and place of living. The survey form included variables related to habitual practices that may impact sleep quality, such as the daily intake of tea or coffee, the amount of time spent using mobile phones, tablets, or laptops, alcohol consumption, smoking habits, and regular exercise [10]. These factors were specifically opted for due to their possible impact on sleep patterns and overall health of individuals. Participants were required to state the frequency and duration of these habits to enhance comprehension of how lifestyle choices might correlate with quality of sleep among medical undergraduates.

The PSQI scale is a validated, self-reported tool commonly used in clinical as well as research settings to evaluate sleep quality over the past one-month period. While various studies have explored the psychometric properties of the scale, the developers' original assessment demonstrated an internal reliability (α) of 0.83, a test-retest reliability of 0.85 for the overall worldwide scale, along with a sensitivity of 89.6% and specificity of 86.5% [12]. The scale encompassed 19 individual items, which are grouped into seven distinct components: subjective sleep quality, sleep latency, sleep duration, habitual sleep efficiency, sleep disturbances, use of sleeping medication, and daytime dysfunction. The questionnaire is an integration of Likert-type and open-ended questions. In scoring the PSQI, seven component scores are calculated, with each score ranging from 0 (no difficulty) to 3 (severe difficulty). These component scores are then added together to generate a global score, which can range from 0 to 21. A PSQI score above 5 reflects poor sleep quality, whereas a score of 5 or below indicates good to excellent sleep quality [12].

Participants were given a specific link to the Google form on a predetermined day. They were asked to complete and submit the form within the decided time period on that day. Before accessing the form, participants were requested to review and provide informed consent, ensuring that they understood the purpose of the study and their voluntary participation. Participants were made clear regarding confidentiality of their responses and the measures in place to protect their personal information.

Data were imported into Microsoft (Redmond, WA, USA) Excel version 2021 for cleaning purposes. IBM SPSS version 26 (IBM Corp., Armonk, NY, USA) was used for analysis. Mean, percentage and standard deviation were used for descriptive inference. Chi-square test of association was used to draw statistical inferences between variables. Fisher's exact test was utilized when expected frequency in any cell of the table was below 5. Binary logistic regression model was used to find out influencing predictors of sleep quality. P value <0.05 is considered statistically significant.

## Results

The mean age of study participants was 20.37 ± 1.47 years. In the present study, 166 (55.4%) female and 134 (44.6%) male students participated. According to phase of studying MBBS, 29.4% of the medical students were from Phase II followed by Phase III part I (26.0%), Phase I (24.0%) and Phase III part II (20.6%). Most of the study participants (58.0%) had normal BMI followed by underweight (21.7%), overweight (14.6%) and obese (5.7%). Almost all (94.6%) study participants were living in a hostel or as a paying guest (Table 1).

**Table 1:** Sociodemographic details of study participants (n = 300) * As per WHO classification of BMI MBBS: Bachelor of Medicine, Bachelor of Surgery

Variable	Frequency (%)
Gender	
Female	166 (55.4)
Male	134 (44.6)
Phase of studying MBBS	
MBBS Phase I	72 (24.0)
MBBS Phase II	88 (29.4)
MBBS Phase III part I	78 (26.0)
MBBS Phase III part II	62 (20.6)
BMI *	
Underweight	65 (21.7)
Normal	174 (58.0)
Overweight	44 (14.6)
Obese	17 (5.7)
Place of living	
Hostel or Paying guest	284 (94.6)
Living with family	16 (5.4)

**Figure 1:**
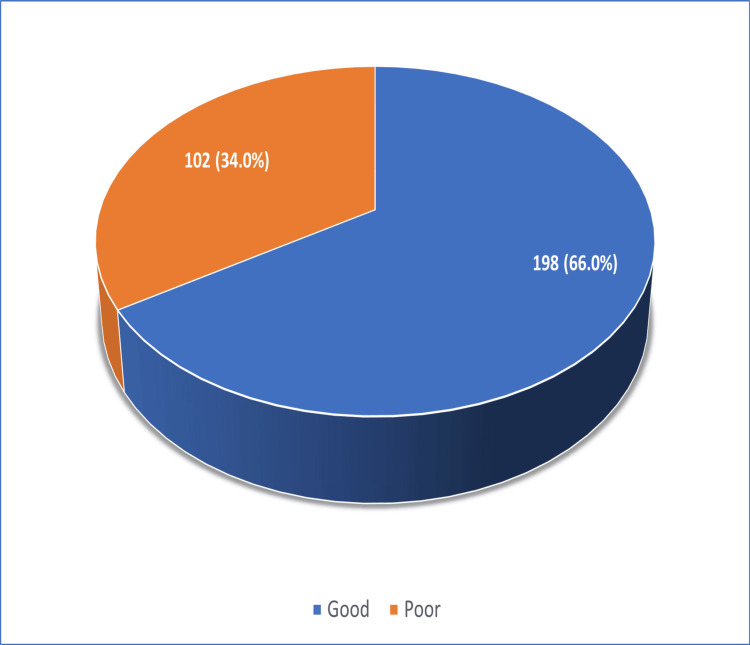
Distribution of study participants according to their sleep quality (n=300)

Sleep quality was poor in 102 (34.0%) of study participants, as depicted in Figure 1.

Almost all study participants (98.0%) were using mobile phones. Nearly one-third of study participants had habits of drinking coffee and laptop/tablet use. Tea intake was observed among 58.0% of study participants. Only half of the study participants (47.3%) were doing exercise. Very few study participants had habits of smoking (3.3%) and alcohol (4.0%) intake (Table 2).

**Table 2:** Habitual practice of the study participants (n = 300)

Habits	Yes (%)	No (%)
Tea intake	174 (58.0)	126 (42.0)
Coffee intake	97 (32.3)	203 (67.7)
Use of Mobile	294 (98.0)	6 (2.0)
Use of Laptop or Tablet	94 (31.3)	206 (68.7)
Alcohol intake	12 (4.0)	288 (96.0)
Smoking	10 (3.3)	290 (96.7)
Exercise	142 (47.3)	158 (52.7)

In the current study, 30.6% of male and 36.8% of female participants had poor quality of sleep, which was not statistically significant (p-value=0.264, X^2^=1.250). Inferior quality of sleep was observed more among MBBS Phase I students (51.4%) followed by MBBS Phase III part II (32.3%). Phase of studying MBBS and quality of sleep had a statistically significant association (p-value=0.004, X^2^=13.440). Among overweight study participants, 50.0% had poor quality of sleep. A statistically significant association was found between BMI and quality of sleep (p-value=0.04, X^2^=6.878). Among the study participants living in a hostel or paying guest, nearly one-third had poor quality of sleep while among those who are residing with their family, nearly one-fourth had poor quality of sleep. There was no statistical association noted between place of living and quality of sleep (p-value=0.435, X^2^=0.610) (Table 3).

**Table 3:** Association between sociodemographic variable and quality of sleep (n=300) * As per WHO classification of BMI MBBS: Bachelor of Medicine, Bachelor of Surgery

Variable	Sleep quality		Total	Chi-square (p – value)
	Good (%)	Poor (%)		
Gender				
Male	93 (69.4)	41 (30.6)	134	1.250 (0.264)
Female	105 (63.2)	61 (36.8)	166	
Phase of studying MBBS				
MBBS Phase I	35 (48.6)	37 (51.4)	72	13.440 (0.004)
MBBS Phase II	63 (71.5)	25 (28.5)	88	
MBBS Phase III part I	58 (74.3)	20 (25.7)	78	
MBBS Phase III part II	42 (67.7)	20 (32.3)	62	
BMI *				
Underweight	42 (64.6)	23 (35.4)	65	6.878 (0.04)
Normal	121 (69.5)	53 (30.5)	174	
Overweight	22 (50.0)	22 (50.0)	44	
Obese	13 (76.4)	4 (23.6)	17	
Place of living				
Hostel or Paying guest	186 (65.4)	98 (34.6)	284	0.610 (0.435)
Living with family	12 (75.0)	4 (25.0)	16	

Of those who had habits of tea intake, coffee intake, use of mobile, use of laptop/tablet, alcohol intake and smoking, about 34% had inferior quality of sleep but there was no statistical association between all these factors and quality of sleep. Of the study participants who exercised regularly, nearly two-thirds had good quality of sleep, although it was not found statistically significant. Of those who had perceived stress during the past month, almost half students (50.8%) had poor quality of sleep, which was highly significant statistically (x^2^ = 27.303, p = 0.000) (Table 4).

**Table 4:** Association between daily habits pattern and quality of sleep (n=300) * Fisher's Exact Test

Variable		Sleep quality		Total	Chi-square (p-value)
		Good (%)	Poor (%)		
Tea intake	Yes	111 (63.7)	63 (36.2)	174	0.899 (0.343)
	No	87 (69.1)	39 (30.9)	126	
Coffee intake	Yes	65 (67.1)	32 (32.9)	97	0.65 (0.798)
	No	133 (65.5)	70 (34.5)	203	
Use of Mobile	Yes	193 (65.6)	101 (34.4)	294	0.820 (0.668)*
	No	5 (83.3)	1 (16.7)	6	
Use of Laptop or Tablet	Yes	57 (60.6)	37 (39.4)	94	1.754 (0.185)
	No	141 (68.4)	65 (31.6)	206	
Alcohol intake	Yes	8 (66.6)	4 (33.4)	12	0.002 (1.000)*
	No	190 (65.9)	98 (34.1)	288	
Smoking	Yes	6 (60.0)	4 (40.0)	10	0.166 (0.739)*
	No	192 (66.2)	98 (33.8)	290	
Exercise	Yes	98 (69.1)	44 (30.9)	142	1.092 (0.296)
	No	100 (63.2)	58 (36.8)	158	
Stressed during past month	Yes	62 (49.2)	64 (50.8)	126	27.303 (0.000)
	No	136 (78.1)	38 (21.9)	174	

Participants with perceived stress during the last month had 4.497 (2.535 - 7.979) higher adjusted odds of poor quality of sleep. Participants in the overweight BMI category had 2.294 (1.033 - 5.095) higher adjusted odds of poor quality of sleep compared to normal BMI category participants. MBBS Phase I students had 2.887 (1.272 - 6.550) higher adjusted odds of poor quality of sleep compared to other phases of studying MBBS students (Table 5).

**Table 5:** Predictors of poor sleep quality: A binary logistic regression model (n=300) MBBS: Bachelor of Medicine, Bachelor of Surgery

Variable	Sig.	Adjusted OR	Lower (CI)	Upper (CI)
Coffee intake	.868	.950	.516	1.747
Tea intake	.338	1.325	.745	2.355
Use mobile	.687	1.609	.159	16.296
Use laptop or tablet	.419	1.288	.697	2.379
Alcohol intake	.335	.399	.062	2.582
Smoking	.392	2.384	.327	17.398
Exercise	.676	.888	.510	1.547
Stressed during the past month	.000	4.497	2.535	7.979
BMI classification (underweight)	.770	.903	.457	1.784
BMI classification (overweight)	.041	2.294	1.033	5.095
BMI classification (obese)	.419	.588	.163	2.127
Gender (female)	.157	1.530	.849	2.759
Place of living (hostel)	.306	1.949	.543	6.996
MBBS Phase I	.011	2.887	1.272	6.551
MBBS Phase II	.635	.823	.367	1.842
MBBS Phase III part I	.264	.620	.269	1.433

## Discussion

Quality of sleep and related disorders are serious concerns that can have long-term sociodemographic implications. In current study, participants with poor sleep quality were 34.0%, while the study done by Binjabr MA et al. had 55.64% participants with poor quality of sleep [13]. About 70% of medical students self-reported sleep deprivation in a study done by Huen LL et al. [14].

We found that there was no observed demarcation in quality of sleep between males and females (p value=0.264, X^2^=1.250). Contrary to that, a study done by Fatima et al. discovered that women were more prone to poor sleep quality and it was found statistically significant as well [15]. On considering academic phases of studying MBBS, a highly significant statistical association was found between phases of studying MBBS and quality of sleep in this study (p value=0.004, X^2^=13.440). A similar finding was observed by Correa CC et al. [16]. MBBS students, especially in Phase 1, face a rigorous curriculum with a vast amount of information to absorb in a relatively short time, moreover, new environment, social adjustment, and academic pressure for good performance sometimes lead to poor quality of sleep [17].

Evidence shows that overweight/obesity and higher residential greenness or place of living are associated with anxiety and poor sleep quality [18]. In our study, nearly half of the overweight medical students had substandard quality of sleep and it was statistically significant (p value=0.04, X^2^=6.878). A similar result was found in a study done by Gupta P et al. [19]. In the current study, no statistically significant association was found between place of living and sleep quality (p-value=0.435, X^2^=0.610). However, a study done by Rezaei et al. observed that students living in their own homes had 2.25 times higher odds of having poor quality of sleep compared to those who were living in a hostel [20].

Poor sleep is one of the health issues significantly associated with heavy alcohol consumption. As per National Health and Nutrition Examination Survey (NHANES) data from 2007-2020 March, smoking and multiple sleep outcomes are significantly associated [21]. Physical activity and physical and mental exercise were found to improve subjective sleep quality in adults mentioned in the study done by Xie et al. [22]. In the present study, we found that there was no statistically relevant connection between quality of sleep and habitual practices like coffee intake (p=0.798, X^2^=0.65), tea intake (p=0.343, X^2^=0.899), alcohol intake (p=1.000., X^2^=0.002) and smoking (p=0.739, X^2^=0.166). Smoking (p = 0.011) and alcohol intake (p=0.048) demonstrated a significant relationship with poor quality of sleep in a study done by Al-Wandi AS et al., which was different compared to our study [23]. Among those who used to exercise, nearly two-thirds had a good quality of sleep in this study. An almost similar finding was noted in a study done by Maat et al. [24].

In this study, we found that there was no meaningful statistical relationship between laptop/tablet (p=0.185, X^2^=1.754) and mobile use (p=0.668, X^2^=0.820) with quality of sleep. A review by Sohn et al. reported that one in every four children and young people are suffering from problematic cell phone use (PSU) and laptop, which is linked to depression, anxiety and poor sleep quality [25]. A study done by Rafique N et al. found that use of laptop or mobile device before bed was positively associated with excessive sleep latency, poor sleep quality, daytime drowsiness and sleep disruptions [26]. In this study, participants who had perceived stress during the last month, nearly half had poor quality of sleep which was statistically significant (p=0.000, X^2^=27.303). A similar finding was observed by Makwana M et al. in their study [27].

A binary logistic regression model was used to find predictors of poor sleep quality. Participants with perceived stress during the last month had 4.497 higher adjusted odds of poor quality of sleep. However, a study done by Alotaibi AD et al. showed a contradictory result (OR = 0.112, 95% CI: 0.035-0.358) [28]. Participants in overweight BMI category had higher adjusted odds of poor quality of sleep compared to normal BMI category participants in our study (adjusted odds ratio (AOR)=2.294,95% CI: 1.033 -5.095). An almost similar finding was found in a study done by Rahe C et al. (AOR= 1.07, CI = 1.01-1.13) [29]. Phase I MBBS students had 2.887 higher adjusted odds of poor quality of sleep compared to other phase students in the current study. A study done by Shrestha D et al. found different results compared to our study (AOR, 0.18; CI, 0.04-0.76) [30].

## Conclusions

Sleep quality was poor in 34.0% of study participants in this study. The academic phase of MBBS plays a crucial role in determining sleep patterns, as early-year students often have more structured schedules, while final-year students face irregular sleep due to exam pressure and clinical responsibilities in this research. The study highlights a strong link between BMI and perceived stress levels with poor quality of sleep among undergraduate MBBS students. Elevated stress and imbalanced BMI may contribute to disrupted sleep patterns, which can negatively impact academic performance and overall well-being.

In the current study, multiple habitual practices were considered (e.g., caffeine intake, screen time, alcohol consumption, smoking, and exercise) to provide a holistic view of factors influencing sleep quality. In this study, binary logistic regression was utilized to identify key predictors of poor sleep quality, allowing for a deeper understanding of influencing factors.

Since this study was performed in a single institution, the results may not reflect the true variability in sleep quality across different academic settings and regions. Larger, multicentric studies are necessary to obtain a more comprehensive understanding. The reported prevalence of sleep disturbances may vary depending on the type of sleep quality assessment tool used, leading to differences in findings across studies.

Medical undergraduates should be provided with workshops on effective time management to balance academic workload, clinical duties, and personal well-being during each phase of MBBS. It is essential for students to adopt stress management strategies such as mindfulness, exercise and time management to alleviate stress levels. Create peer support groups for students to openly talk about their sleep, stress and well-being challenges with their peers to build a community. Optimal BMI (a combination of a well-balanced diet and regular active lifestyle) can also increase desire for sleep. Regular physical exercise through fitness classes or organised sports, meditation, yoga, etc. can reduce stress and improve sleep quality. These efforts might also play a vital role in uplifting the mental health of future healthcare professionals. 

## References

[1] Kline CE, Sanders RM, Cheruka CA (2024). Associations between reallocations of daytime movement behaviors and self-reported sleep quality and insomnia severity in desk workers. Circulation.

[2] Pilcher JJ, Ott ES (1998). The relationships between sleep and measures of health and well-being in college students: a repeated measures approach. Behav Med.

[3] Cartwright RD (1991). Development of a program for sleep disorders. Handbook of Clinical Psychology in Medical Settings.

[4] Ferrara M, De Gennaro L (2001). How much sleep do we need?. Sleep Med Rev.

[5] Rosen RC, Rosekind M, Rosevear C, Cole WE, Dement WC (1993). Physician education in sleep and sleep disorders: a national survey of U.S. medical schools. Sleep.

[6] Yang CM, Wu CH, Hsieh MH, Liu MH, Lu FH (2003). Coping with sleep disturbances among young adults: a survey of first-year college students in Taiwan. Behav Med.

[7] Orzech KM, Salafsky DB, Hamilton LA (2011). The state of sleep among college students at a large public university. J Am Coll Health.

[8] Verlander LA, Benedict JO, Hanson DP (1999). Stress and sleep patterns of college students. Percept Mot Skills.

[9] Altena E, Baglioni C, Espie CA (2020). Dealing with sleep problems during home confinement due to the COVID-19 outbreak: practical recommendations from a task force of the European CBT-I Academy. J Sleep Res.

[10] Jasani P, Chauhan D, Makwana M, Patel U (2023). Prevalence of major depressive disorder and its associated factors among students of private higher secondary schools of science stream, Rajkot city, Gujarat: a cross-sectional study. Natl J Physiol Pharm Pharmacol.

[11] Binjabr MA, Alalawi IS, Alzahrani RA (2023). The worldwide prevalence of sleep problems among medical students by problem, country, and COVID-19 status: a systematic review, meta-analysis, and meta-regression of 109 studies involving 59427 participants. Curr Sleep Med Rep.

[12] Buysse DJ, Reynolds CF 3rd, Monk TH, Berman SR, Kupfer DJ (1989). The Pittsburgh Sleep Quality Index: a new instrument for psychiatric practice and research. Psychiatry Res.

[13] Azad MC, Fraser K, Rumana N, Abdullah AF, Shahana N, Hanly PJ, Turin TC (2015). Sleep disturbances among medical students: a global perspective. J Clin Sleep Med.

[14] Huen LL, Chan TW, Yu WM, Wing YK (2007). Do medical students in Hong Kong have enough sleep?. Sleep Biol Rhythms.

[15] Fatima Y, Doi SA, Najman JM, Mamun AA (2016). Exploring gender difference in sleep quality of young adults: findings from a large population study. Clin Med Res.

[16] Corrêa CC, Oliveira FK, Pizzamiglio DS, Ortolan EV, Weber SA (2017). Sleep quality in medical students: a comparison across the various phases of the medical course. J Bras Pneumol.

[17] Periasamy P, Vajiravelu S, Gunasekaran S, Balakrishnan R, Manivannan J (2022). Association between sleep quality and stress among medical students of a rural indian tertiary care setting. MGM J Med Sci.

[18] Dakanalis A, Voulgaridou G, Alexatou O (2024). Overweight and obesity is associated with higher risk of perceived stress and poor sleep quality in young adults. Medicina (Kaunas).

[19] Gupta P, Srivastava N, Gupta V, Tiwari S, Banerjee M (2022). Association of sleep duration and sleep quality with body mass index among young adults. J Family Med Prim Care.

[20] Rezaei O, Mokhayeri Y, Haroni J (2017). Association between sleep quality and quality of life among students: a cross sectional study. Int J Adolesc Med Health.

[21] Sun H, Li S (2024). Exploring the relationship between smoking and poor sleep quality: a cross-sectional study using NHANES. Front Psychiatry.

[22] Xie W, Lu D, Liu S, Li J, Li R (2024). The optimal exercise intervention for sleep quality in adults: a systematic review and network meta-analysis. Prev Med.

[23] Al-Wandi AS, Shorbagi SI (2020). Sleep patterns and its relation to lifestyle habits: a study of secondary high school students in Sharjah, United Arab Emirates. AIMS Public Health.

[24] Alnawwar MA, Alraddadi MI, Algethmi RA, Salem GA, Salem MA, Alharbi AA (2023). The effect of physical activity on sleep quality and sleep disorder: a systematic review. Cureus.

[25] Sohn SY, Rees P, Wildridge B, Kalk NJ, Carter B (2019). Prevalence of problematic smartphone usage and associated mental health outcomes amongst children and young people: a systematic review, meta-analysis and GRADE of the evidence. BMC Psychiatry.

[26] Rafique N, Al-Asoom LI, Alsunni AA, Saudagar FN, Almulhim L, Alkaltham G (2020). Effects of mobile use on subjective sleep quality. Nat Sci Sleep.

[27] Makwana M (2022). Prevalence and determinants of stress among undergraduate medical students in Surendranagar district: a cross-sectional study. Natl J Physiol Pharm Pharmacol.

[28] Alotaibi AD, Alosaimi FM, Alajlan AA, Bin Abdulrahman KA (2020). The relationship between sleep quality, stress, and academic performance among medical students. J Family Community Med.

[29] Rahe C, Czira ME, Teismann H, Berger K (2015). Associations between poor sleep quality and different measures of obesity. Sleep Med.

[30] Shrestha D, Adhikari SP, Rawal N (2021). Sleep quality among undergraduate students of a medical college in Nepal during COVID-19 pandemic: an online survey. F1000Res.

